# The *Modus Operandi* of Hydrogen Sulfide(H_2_S)-Dependent Protein Persulfidation in Higher Plants

**DOI:** 10.3390/antiox10111686

**Published:** 2021-10-26

**Authors:** Francisco J. Corpas, Salvador González-Gordo, María A. Muñoz-Vargas, Marta Rodríguez-Ruiz, José M. Palma

**Affiliations:** Group of Antioxidants, Free Radicals, and Nitric Oxide in Biotechnology, Food and Agriculture, Department of Biochemistry, Cell and Molecular Biology of Plants, Estación Experimental del Zaidín, Spanish National Research Council, CSIC, C/Profesor Albareda, 1, 18008 Granada, Spain; salvador.gonzalez@eez.csic.es (S.G.-G.); mangeles.munoz@eez.csic.es (M.A.M.-V.); marta.rodriguez@eez.csic.es (M.R.-R.); josemanuel.palma@eez.csic.es (J.M.P.)

**Keywords:** hydrogen sulfide, persulfidation, oxidative posttranslational modifications, *S*-desulfurization

## Abstract

Protein persulfidation is a post-translational modification (PTM) mediated by hydrogen sulfide (H_2_S), which affects the thiol group of cysteine residues from target proteins and can have a positive, negative or zero impact on protein function. Due to advances in proteomic techniques, the number of potential protein targets identified in higher plants, which are affected by this PTM, has increased considerably. However, its precise impact on biological function needs to be evaluated at the experimental level in purified proteins in order to identify the specific cysteine(s) residue(s) affected. It also needs to be evaluated at the cellular redox level given the potential interactions among different oxidative post-translational modifications (oxiPTMs), such as *S*-nitrosation, glutathionylation, sulfenylation, *S*-cyanylation and S-acylation, which also affect thiol groups. This review aims to provide an updated and comprehensive overview of the important physiological role exerted by persulfidation in higher plants, which acts as a cellular mechanism of protein protection against irreversible oxidation.

## 1. H_2_S Metabolism in Higher Plants: A Perspective

**Figure 1:**
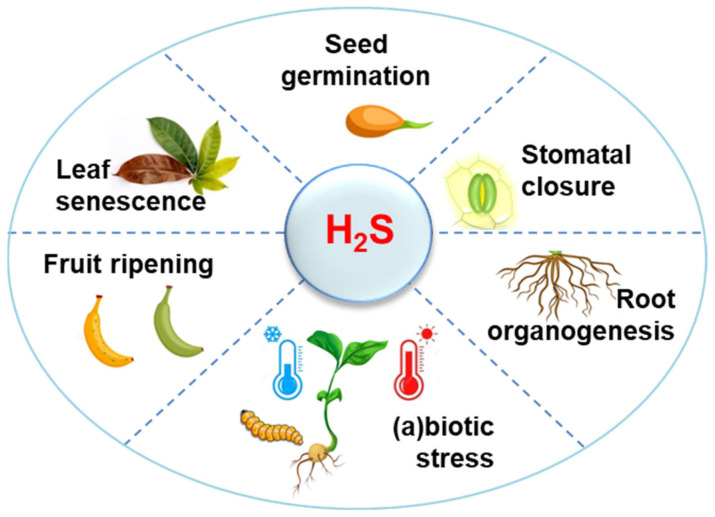
Main plant physiological and stressful processes where hydrogen sulfide (H_2_S) is involved. All these wide spectra of functions exerted by H_2_S are done usually in coordination with other molecules with regulatory properties such as nitric oxide, hydrogen peroxide, carbon monoxide, melatonin, abscisic acid, gibberellins, cytokinins, or ethylene.

Hydrogen sulfide (H_2_S) is currently recognized as a new signaling molecule, with cytoprotective properties similar to those of gasotransmitters such as nitric oxide (NO) and carbon monoxide (CO). The consensus surrounding the biological function of H_2_S was established 25 years ago through Professor Hideo Kimura’s pioneer animal systems research group. They discovered that H_2_S is endogenously generated and acts as a brain neuromodulator [1] whereas later was found to regulate smooth muscle tone [2]. It is important to note that the effect of exogenous H_2_S in higher plants was explored and reported even before its impact on mammalian cells was described. For example, in 1946, Petersen [3] reported that pre-treatment of fresh vegetables, such as cabbage (*Brassica oleracea*), kale (*Brassica oleracea* var. sabellica) and parsley (*Petroselinum crispum*), with gaseous H_2_S has a marked effect on ascorbic acid content retention during hot air drying. In 1975, Joshi et al. [4] showed that exogenous H_2_S inhibits the respiration and oxidative power of rice (*Oryza sativa*) roots and also affects a number of physiological parameters with respect to different rice cultivars. However, research into its role in higher plant physiology has been increasing to a significant extent particularly over the last ten years. H_2_S is now reported to be involved in virtually all physiological processes, including seed germination, root development, senescence, fruit ripening and guard cell movements [5,6,7,8], and also in mechanisms of response to adverse environmental conditions, as well as to biotic and abiotic stresses [9,10,11,12,13,14,15,16,17,18,19]. Figure 1 shows a graphical summary of the key plant processes involving H_2_S.

At the metabolic level, H_2_S is part of the cysteine (Cys) metabolism which is present in different subcellular compartments such as cytosol, plastids and mitochondria [20,21,22,23] and references therein]. More recently, peroxisomes have been reported to be involved in the H_2_S metabolism, with certain peroxisomal enzymes found to be targeted by persulfidation [6]. However, the potential enzymatic source of this PTM in these organelles has not yet been identified.

H_2_S is endogenously generated in the cellular cysteine metabolism and is no longer regarded by the scientific community as a harmful by-product, but rather as a signal molecule, with the capacity to regulate the cellular metabolism, either by itself or in cooperation with other signal molecules such as nitric oxide (NO) [24,25].

## 2. Persufidation: A Redox Regulation of Thiol Groups

Cysteine thiols, considered to be redox-active switch residues, are highly nucleophilic due to their large S atom radius and the low dissociation energy of the thiol S-H bond [26,27]. The reactivity of the thiol group to electrophilic molecules, such as certain reactive oxygen/nitrogen species (ROS/RNS), increases following ionization. Furthermore, the importance of Cys residues is explained by their location in the catalytic active site of the protein, metal ligation and their involvement in protein tertiary structure stability, thus indicating that these residues are involved in numerous regulatory protein functions.

Protein-bound thiol groups are subject to complex redox regulation. This depends on the equilibrium of all the thiol-bearing compounds involved, including proteins, peptides, such as the reduced/oxidized glutathione couple (GSH/GSSG) and free cysteine/cystine (C/CSSC), as well as on cellular compartment localization, in addition to physiological and stress status.

**Figure 2:**
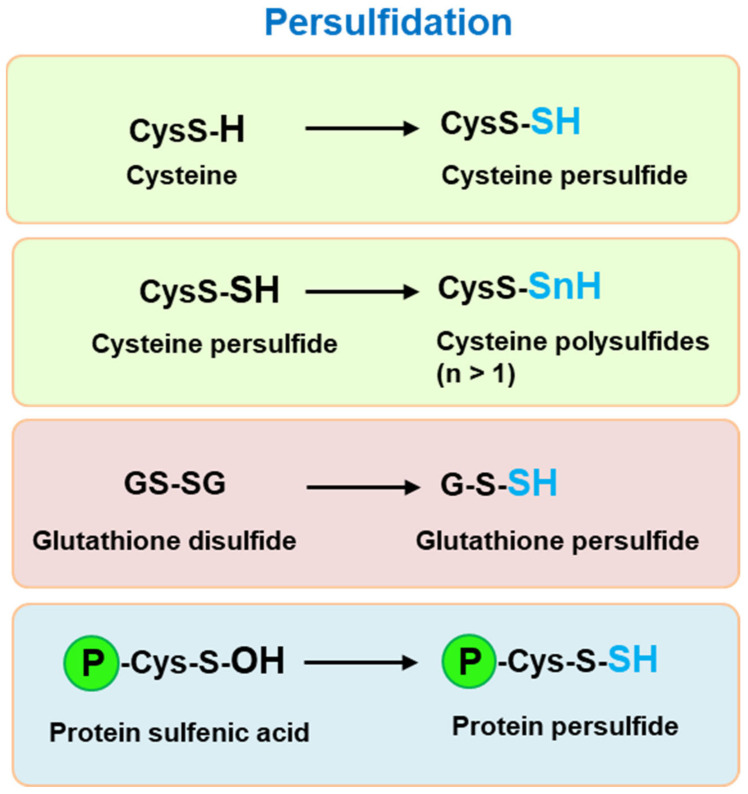
Examples of persulfidation of low and high molecular weight thiol compounds including cysteine (Cys), glutathione disulfide (GSSG) and protein undergoing Cys modifications (P).

Persulfidation, previously called *S*-sulfhydration, is a reversible oxiPTM, in which the cysteine thiol group (-SH) is transformed into its corresponding persulfide form (-SSH). This process involves the following possible reactions: i) between a protein thiol and H_2_S, specifically with its anionic hydrosulfide form (HS^–^) which is a stronger nucleophilic agent than its protonated form; ii) between inorganic polysulfides (-S-Sn-S-) and protein thiolates; and iii) a radical reaction to other reactive sulfur species (RSS). A trans-persulfidation reaction among molecules such as glutathione persulfide (GSSH), cysteine persulfide (Cys-SSH) and protein persulfides, together with the involvement of GSH and Cys, may also occur [28,29,30,31,32]. Figure 2 shows some examples of the persulfidation of low and high molecular weight thiol compounds.

In mammalian cells, a novel cysteine persulfide synthase, cysteinyl-tRNA synthetase (CARS), has been identified. CARS is capable of generating Cys-SSH and polysulfides which are involved in the regulation of mitochondrial biogenesis and the bioenergetic metabolism [33,34] and also provide cytoprotection against oxidative stress [35]. However, to the best of our knowledge, this cysteine persulfide synthase has not been found in plant cells.

## 3. Protein Persulfidation and Other Cysteine Modifications

Over the last ten years, the development of new mass spectrometry (MS)-based high-throughput proteomic techniques has led to an increase in the identification of proteins susceptible to persulfidation. In spinach (*Spinacia oleracea*) plants exposed to 100 µM sodium hydrosulfide (NaHS), an H_2_S donor, proteomic analysis of leaves has shown how H_2_S affects protein expression. Thus, of the 1000 proteins identified, 92 were differentially expressed (an increase of over 2-fold), 65 were up-regulated and 27 down-regulated due to the treatment [36]. On the other hand, using the biotin switch method, suitably adapted in order to identify persulfidated proteins, combined with liquid chromatography-tandem mass spectrometry (LC-MS/MS) analysis, a total of 106 putative proteins targeted for persulfidation were identified in *Arabidopsis thaliana* leaves [37]. Subsequently, using an improved tag-switch method, these authors expanded the number of persulfidated proteins to 2015 [38]. More recently, the number of persulfidated proteins in arabidopsis roots has reached 5214 through the use of extremely high-resolution mass spectrometer [39]. These plant proteomic studies that identified potential protein targets of persulfidation were used as the starting point for complementary analyses to specifically identify the residue(s) persulfidated and to determine the effect of this oxiPTM on the function of the proteins targeted [40]. Table 1 [41,42,43,44,45,46,47,48,49,50,51,52,53] shows an updated list of the plant proteins undergoing persulfidation, and the effect on protein function. These persulfidated proteins are involved in a wide range of cellular processes, including photosynthesis, the sulfur metabolism, reactive oxygen/nitrogen species (ROS/RNS) activity, ethylene biosynthesis, organelle movements, autophagy and ABA signaling, thus confirming the important role of H_2_S in plant cell physiology.

It is worth noting that, following NaHS treatment, the H_2_S-generating enzyme, L-cysteine desulfhydrase1 (DES1), is persulfidated in Cys44 and Cys205 and subsequently upregulated [42]. Likewise, respiratory burst oxidase homolog protein D (RBOHD), which is involved in stomatal movements, is also persulfidated in Cys825 and Cys890 and its activity is upregulated [42].

In *Arabidopsis thaliana*, 3-mercaptopyruvate sulfurtransferase (MST), specifically the sulfurtransferase 1 and sulfurtransferase 2 (STR1 and STR2), has recently been shown to mediate either protein persulfidation or H_2_S formation, as well as low molecular weight persulfide formation [54]. *S*-desulfurization, a new GSH-mediated process, as opposed to persulfidation, involving the release of H_2_S from persulfidated proteins, has also been reported. *S*-desulfurization mitigates the inhibition by persulfidation of various enzymes including alliinase, D-lactate dehydrogenase (D-LDH), alcohol dehydrogenase (ADH) and glucose-6-phosphate dehydrogenase (G6PDH) [55]. These findings provide further confirmation of the important role played by persulfidation in the regulation of its target proteins.

**Figure 3:**
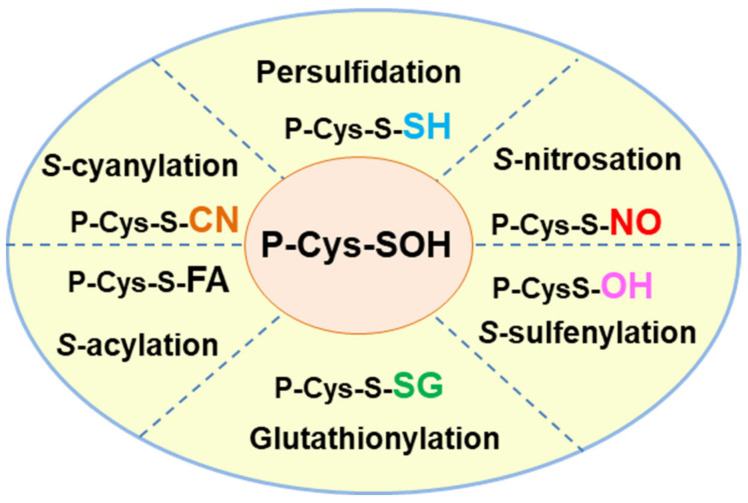
Hallmark of the different oxidative posttranslational modifications (oxiPTMs) of cysteine-containing proteins including persulfidation, *S*-nitrosation, *S*-sulfenylation, glutathionylation, *S*-acylation and *S*-cyanylation which are mediated by hydrogen sulfide (H_2_S), nitric oxide (NO), hydrogen peroxide (H_2_O_2_), glutathione (GSH), fatty acid (FA) and cyanide (CN^-^), respectively. The thiol group (-SH) of Cys residues works as a redox switch and each of the PTMs can modify the function of the target protein either positively or negatively.

In addition to persulfidation, other competing PTMs can affect thiol groups. Figure 3 shows a summary of the principal oxiPTMs in which protein thiol groups are involved. *S*-glutathionylation is mediated by the addition of the tripeptide glutathione (GSH) γ-L-glutamyl-L-cysteinylglycine [56], an antioxidant molecule which is one of the most abundant low molecular mass thiols in plant cells [57]. GSH also interacts with NO to form *S*-nitrosoglutathtione (GSNO) which is regarded as a NO reservoir [58]. *S*-sulfenylation, which results from the oxidation of the thiol group to Cys sulfenic acid, is mediated by H_2_O_2_ [59,60]. *S*-acylation is a reversible oxiPTM involving the addition of a fatty acid, such as palmitate or stearate, to specific cysteines through thioester bonds [61,62]. *S*-cyanylation is a new PTM mediated by cyanide (CN^-^) [63] which opens up a whole new range of functions that need to be explored. Recently, an in silico platform named pCysMod was set up to predict multiple Cys PTMs [64], such as *S*-nitrosation, *S*-glutathionylation, *S*-cyanylation, *S*-sulfenylation and S-acylation, due to interactions with NO, GSH, cyanide, hydrogen peroxide (H_2_O_2_) and fatty acids, respectively. The integrative database iCysMod (http://icysmod.omicsbio.info/#; accessed on 26 October 2021) for protein cysteine modifications (PCMs) in 48 eukaryotes including *Arabidopsis thaliana* has also been established [65]. This database is a useful tool for investigating the mechanisms underlying the regulatory processes involving these oxiPTMs.

In higher plants, ascorbate peroxidase (APX) constitutes a good example of a protein whose Cys residues are affected by oxiPTMs. This key antioxidant enzyme, which is present in virtually all subcellular compartments, is a component in the ascorbate-glutathione cycle [66,67,68,69,70]. APX catalyzes the decomposition of H_2_O_2_ associated with the oxidation of ascorbate to dehydroascorbate. Various studies have shown that, in a diverse range of plant species, APX is targeted by oxiPTMs including glutathionylation [69], *S*-nitrosation [71,72] (Begara-Morales et al. 2014; Hu et al. 2015), *S*-persulfidation [37], *S*-sulfenylation [59] and *S*-cyanylation [63] (García et al., 2019). While APX activity is prone to sulfenylation involving inactivation by critical cysteine oxidation, the other PTMs appear to act protectively. Catalase (CAT), the principal H_2_O_2_-catalyzing enzyme in eukaryotes, is also susceptible to post-translational modification by oxidation, nitration, *S*-nitrosylation and persulfidation [6,73]. CAT, which, with its high Km reaction rate, dismutates hydrogen peroxide, as well as APX, which fine-tunes CAT’s enzymatic role, acts synergistically to control H_2_O_2_ levels. This diversity of regulatory events points to how all these oxyPTMs modulate H_2_O_2_ content in different subcellular compartments, particularly in peroxisomes, where CAT and APX are present. There are other examples such as some NADPH generating enzymes, i.e., NADP-glyceraldehyde-3-phosphate dehydrogenase and NADP-isocitrate dehydrogenase, which can undergo some of these oxiPTMs (persulfidation and *S*-nitrosation) affecting its capacity to generate NADPH and, consequently, modulate the redox state of the cell [74].

## 4. Conclusions and Future Perspectives

The important role of H_2_S in the physiology of higher plants is now widely recognized, with the H_2_S-based PTM protein persulfidation being particularly effective at modulating the function of target proteins. However, other underexplored H_2_S-related molecules in the plant metabolism such as glutathione persulfide (GSSH) and cysteine persulfide (CysSSH) could increase the biochemical role of H_2_S in protein functions through trans-persulfidation and by regulating cellular redox state. Furthermore, it is important to note that data obtained under in vitro conditions using an H_2_S donor are very useful for determining which Cys residues are potential targets of H_2_S generated PTMs in order to evaluate their positive or negative effects under controlled conditions. However, this information may occasionally contradict results obtained in vivo under cellular redox conditions where different molecules compete with one another for specific residues. Thus, research needs to be carried out in the future on the important role played by H_2_S and on its interactions with other macromolecules in order to gain new insights into the exact modes of action of this gasotransmitter.

## Figures and Tables

**Table 1:** Identified plant protein targets of persulfidation whose function is positive or negatively affected by H_2_S.

Enzyme/Protein	Function	Effect	Ref.
RuBISCO	Photosynthesis	Activity up-regulated	[41]
O-acetylserine(thiol)lyase (OAS-TL)	Sulfur metabolism	Activity up-regulated	[41]
L-cysteine desulphydrase (LCD)	Sulfur metabolism	Activity up-regulated	[42]
Ascorbate peroxidase (APX)	Antioxidant	Activity up-regulated	[14,37]
Glyceraldehyde 3-phosphate dehydrogenase (GAPDH)	Production of energy in the glycolysis	Activity up-regulated	[37]
Glutamine synthetase (GS)	Metabolism of nitrogen	Activity down-regulated	[37]
Actin	Organelle movement, cell division and expansion	Inhibits actin polymerization	[43]
1-aminocyclopropane-1-carboxylic acid oxidase (ACO)	Ethylene biosynthesis	Activity down-regulated	[44]
NADP-isocitrate dehydrogenase (NADP-ICDH)	Provides NADPH as a reducing agent	Activity down-regulated	[45]
NADP-malic enzyme (NADP-ME)	Provides NADPH as a reducing agent	Activity down-regulated	[46]
Catalase	Antioxidant	Activity down-regulated	[6,14]
SNF1-RELATED PROTEIN KINASE2.6 (SnRK2.6)	Promotes ABA signaling	Promotes stomatal closureActivity up-regulated	[47,48]
Respiratory burst oxidase homolog protein D (RBOHD)	Generation of superoxide radical	Activity up-regulated	[42]
Cysteine protease ATG4	Autophagy	Inhibits autophagy	[49]
ATG18	Autophagy	Negative regulation	[50]
Peroxidase	ROS metabolism	Activity up-regulated	[14]
Flowering Locus C protein (FLC1 and 3)	Flowering regulatory pathway	Reduces binding abilities of FLCs	[51]
Mitogen-activated protein kinase (MPK) 4	Allivates cold stress	Activity up-regulated	[52]
Abscisic acid insensitive 4 (ABI4)	Regulates ABA signaling	Enhanced the transactivation activity of ABI4 towards MAPKKK18	[53]
